# Hyperbaric Oxygen Therapy in Ophthalmology: A Narrative Review

**DOI:** 10.3390/jcm13010029

**Published:** 2023-12-20

**Authors:** Zuzanna Micun, Weronika Dobrzyńska, Michał Sieśkiewicz, Izabela Zawadzka, Diana Anna Dmuchowska, Marzena Wojewodzka-Zelezniakowicz, Joanna Konopińska

**Affiliations:** 1 Faculty of Medicine, Medical University of Białystok, Jana Kilinskiego 1 STR, 15-089 Białystok, Poland; 36297@student.umb.edu.pl (Z.M.);; 2 Department of Ophthalmology, Medical University of Bialystok, Jana Kilinskiego 1 STR, 15-089 Białystok, Poland; okulistyka@umb.edu.pl (W.D.); diana.dmuchowska@umb.edu.pl (D.A.D.); 3 Department of Emergency and Disaster Medicine, Medical University of Bialystok, Jana Kilinskiego 1 STR, 15-089 Białystok, Poland; marzena.wojewodzka-zelezniakowicz@umb.edu.pl

**Keywords:** acute optic neuropathy, retinal artery occlusion, choroidal circulation, hyperbaric oxygen therapy (HBOT), macular edema

## Abstract

Hyperbaric oxygen therapy (HBOT) has been used for the past 50 years for conditions such as decompression disease and wound healing. It has promising effects in the treatment of vision-threatening diseases, such as retinal artery occlusion, retinal vein occlusion, diabetic macular edema, and acute optic neuropathy; however, HBOT has not been approved for use in these conditions by regulatory authorities. This paper provides an overview of the theoretical effectiveness and most recent indications for HBOT in ophthalmology. The fundamental aspects of the physiology of choroidal circulation and metabolism are provided together with the clinical aspects that should be accounted for when selecting patients for this therapy. The paper also presents case reports of when HBOT was successfully implemented. The goals of this review were to explore the indications and benefits of HBOT and to evaluate the effectiveness of HBOT as an intervention in treating ophthalmology disorders. Lastly, the paper details the side-effects and discusses the safety issues of HBOT.

## 1. Introduction

The retina has the highest rate of oxygen consumption among all organs; thus, hypoxic-ischemic damage is frequently observed in many ophthalmic diseases. Hyperbaric oxygen therapy (HBOT), a medical treatment that aims to increase the levels of dissolved oxygen in tissues, has been used as adjuvant management of vision-threatening diseases, such as retinal artery occlusion (RAO), retinal vein occlusion (RVO), diabetic macular edema (DME), and acute optic neuropathy; however, HBOT has not been approved for use in these conditions by regulatory authorities. During HBOT, patients breathe nearly 100% oxygen inside a hyperbaric chamber pressurized to 1 atmosphere absolute (ATA). Nitrogen in the vitreous is replaced with oxygen during the therapy and the vitreous acts as an oxygen reserve during the first 2 days of treatment. Tissue oxygen level has been observed to remain high for up to 4 h after the therapy [1]. The administration of oxygen at high atmospheric pressure increases oxygen transport into the tissues, and the use of intraocular HBOT in ischemic ophthalmic disorders has been shown to increase the oxygen saturation of the vitreous body. HBOT protects the injured retinal neuronal cells from apoptosis [2]. Based on the results observed in experimental studies in animal models, similar beneficial effects of HBOT can be observed in the treatment of human retinal ischemia [3]. HBOT can also be used to achieve a higher diffusion gradient from the choroidal circulation to the outer layers [4]. However, HBOT is underutilized in ophthalmology, given the poor-quality evidence supporting the use of HBOT as a treatment, and all its indications are currently off-label.

Few studies have explored the usefulness of HBOT in ophthalmic diseases. In 2008, Butler et al. [5] comprehensively reviewed the role of HBOT in ocular disorders by reviewing nine ocular disorders indicated to receive HBOT by the Undersea and Hyperbaric Medical Society, including decompression sickness or arterial gas embolism and carbon monoxide poisoning with visual symptoms. Other ocular indications for HBOT that are supported by considerable evidence include ischemic optic neuropathy, retinal artery/vein occlusion, and macular edema. Their review suggested that the effectiveness of HBOT in these disorders varies owing to differences in the time interval between the onset of symptoms and initiation of HBOT. However, numerous studies have demonstrated the efficacy and versatility of hyperbaric therapy in ophthalmology since the publication of this article [3,6,7,8,9,10,11,12]. Our review provides an updated overview of the most recent indications for HBOT in ophthalmology.

### Search Methodology

The objective of this study was to conduct a comprehensive review to assess the efficacy of hyperbaric oxygen therapy (HBOT) in the treatment of ophthalmological diseases. A systematic search of relevant literature was performed across multiple electronic databases, including EMBASE, PubMed, ScienceDirect, Web of Science, The Cochrane Library, Google Scholar, and Ovid MEDLINE. The search encompassed various combinations of keywords, such as “HBOT and CRAO/BRAO” (central retinal artery occlusion/branch retinal artery occlusion), “HBOT and retina”, “HBOT and eye”, “HBOT and macula”, “HBOT and diabetic retinopathy”, “HBOT and cornea”, and “HBOT and optic neuropathy”. The latest search was conducted on 11 September 2023. Additional relevant studies were identified by manually searching the reference lists of the retrieved publications. The inclusion criteria comprised original studies, case reports, systematic reviews, and meta-analyses published in English. To avoid duplication, if the same study population was presented in multiple publications, the most recent one was considered for eligibility. Exclusions were made for self-standing abstracts, articles with duplicated data, and those lacking original data. Publications not meeting specific research criteria or lacking clear methods were also excluded. Data extraction involved two independent authors (Z.M. and W.D.) who gathered information on country, first author, publication year, study population details, intervention, study design, and follow-up duration. Any inconsistencies were resolved through consensus discussions.

## 2. Impact of HBOT on Healthy Retinas

The retinal artery (RA) and choroid are the two main sources of blood supply to the retina. Diffusion from the choroidal circulation (CC) provides nourishment to the outer retina and the foveal avascular zone (FAZ). Although the internal autoregulatory system of the retina alters the blood flow and vascular density in response to unexpected conditions to maintain blood flow and oxygenation stability [13], it may not function correctly under conditions such as diabetic retinopathy (DR) or age-related macular degeneration [14,15]. Macular microvasculature is a complex system comprising three capillary plexuses for the blood supply of the inner retina: the superficial retinal plexus (SP) located in the retinal nerve fiber layer and the two plexuses located at the inner and outer borders of the inner nuclear layer, which constitute the deep retinal plexus (DP) [16]. It has been observed that responses to hypoxia in RA circulation and CC vary [17].

Recent studies have evaluated what impact HBOT has on the anatomical features of a healthy retina using modern imaging techniques, such as spectral domain optical coherence tomography (SD-OCT) and OCT angiography [6,18,19]. However, their results are contradictory.

A recent study by Sayin et al. [6] investigated the effects of HBOT on the retinal layers in healthy eyes. The study group consisted of 30 patients: 14 female and 16 male patients. The indications for HBOT were as follows: sudden hearing loss in 21 patients (70%), chronic osteomyelitis in two patients (6.7%), and aseptic necrosis in seven patients (23.3%). The control group consisted of nine female and 11 male patients. SD-OCT was performed to obtain automated measurements of the thickness of each retinal layer. Retinal OCT scans were performed before HBOT and after the first and tenth sessions, and the results were compared between the visits. No changes were observed in the thickness of the retinal layers after the first and tenth sessions in healthy eyes. This result is not consistent with that of the study by Tukenmez Dikmen et al. [19], who evaluated the effect of HBOT on the vascular and stromal components of the choroid and macula in healthy eyes. They performed SD-OCT before and half an hour after the first and twentieth sessions of HBOT and demonstrated a statistically significant change in the central macular thickness before and after HBOT. Thus, it was hypothesized that HBOT might be considered an adjunctive and facilitating treatment option, in addition to current treatments, for patients with macular edema resulting from retinal vascular disorders.

Çevik et al. [18] reported that a healthy retina responds to HBOT oxygen supersaturation, leading to a decrease in the vascular density of all layers. Notably, they also reported significant decreases in SP and DP capillary network densities in the macular region. However, there was no effect on the FAZ measurements, and it was emphasized that HBOT increases the oxygen concentration in the avascular outer retina via increased supply from the CC, which maintains constant flow at high oxygen levels. DP must counteract greater vasoconstriction to maintain a steady oxygen flux in the outer retina.

## 3. HBOT in Retinal Diseases

### 3.1. DR and DME

Diabetes mellitus is one of the fastest growing and most prevalent causes of morbidity and mortality worldwide [20]. It is characterized by chronic hyperglycemia, which can lead to various systemic vascular complications, such as stroke, cardiovascular disease, peripheral artery disease, and microangiopathies (including nephropathy, neuropathy, chronic diabetic foot ulcers, and retinopathy). DR and DME are considered the most common chronic microvascular complications of diabetes mellitus, which can lead to vision impairment and a decrease in the quality of life among working-age adults [12]. Based on the assessment of microvascular lesions on the retina, DR can be classified into two stages: non-proliferative diabetic retinopathy (NPDR) and the more severe proliferative diabetic retinopathy (PDR). NPDR is characterized by the presence of microaneurysms, intraretinal hemorrhages, venous beading, cotton wool spots, hard exudates, and intraretinal microvascular anomalies. In contrast, PDR is characterized by neovascularization (NV), which is the formation of new and abnormal blood vessels in the retina, optic disc, and iris, and it can lead to vision loss due to vitreous hemorrhage, tractional retinal detachment, and glaucoma. Notably, visual acuity (VA) can deteriorate at any stage of retinopathy regardless of the presence of DME.

Microvascular occlusion, the breakdown of the blood-retinal barrier leading to capillary leakage, and chronic local inflammation are considered the main mechanisms of DR. However, recent studies have shown that dysfunction of the neurovascular unit of the retina contributes to the pathogenesis of DR [7]. The pathophysiological role of diabetic choroidopathy cannot be neglected. Understanding the pathogeneses of DR and DME has become crucial for the development of new therapies. Laser photocoagulation and intravitreal pharmacological agents, such as vascular endothelial-derived growth factor (VEGF) pathway inhibitors and corticosteroids, have revolutionized the treatment of DR and DME. Nevertheless, developing novel treatments for patients who do not respond to available therapies remains crucial.

For over three decades, researchers have speculated that HBOT may be beneficial in the treatment of DR. However, there is no conclusive evidence regarding the effect of HBOT on the course of DR in the literature. Previous experimental studies have demonstrated that HBOT ameliorates the breakdown of the blood-retinal barrier; thus, it may lead to the assumption that HBOT can treat macular edema [21]. However, there have been only a few preliminary reports on the positive impact of HBOT on DME [22,23].

Sellman et al. [9] evaluated the effects of HBOT on VA and retinopathy in patients with chronic diabetic foot ulcers through a prospective, randomized, double-blind, placebo-controlled study with a 2-year follow-up period. The 50 patients included in their study were divided into two groups, and the outcomes of the HBOT group were compared with those of the placebo group. No differences in VA, stage of retinopathy, or macular edema were observed between the two groups. Thus, treatment with HBOT was considered neutral from an ophthalmological perspective.

These results differ considerably from those reported by Maalej et al. [24], who demonstrated a reduction in central macular thickness (CMT) and stabilization of DR lesions in patients who received HBOT. In their study, patients with NPDR were divided into two groups of 25 patients each. The first group received HBOT for 6 weeks as a treatment for diabetic foot ulcers, whereas the second group was followed up without HBOT. Patients did not receive concurrent ophthalmic treatments. Ophthalmic examinations and SD-OCT performed before and 6 weeks after receiving therapy revealed that HBOT could be used as an adjunct treatment for the management of retinal ischemia and capillary hyperpermeability in DR.

Similar results were obtained in a prospective, non-randomized cohort study conducted by Kaldırım et al. [25]. In their study, patients with type 2 diabetes and coexisting chronic diabetic foot ulcers were divided into the following three adjuvant therapy groups: (1) mild-to-moderate NPDR, (2) severe NPDR, and (3) PDR without active proliferative findings that had received applied laser therapy for at least 2 years. All participants received 30 sessions of HBOT for the treatment of diabetic foot ulcers. Ophthalmic examination and OCT scans were performed before treatment, after the tenth, twentieth, and thirtieth sessions, and 10 days after completing the treatment. Although no progression or regression of the PDR stage was observed in any group, HBOT resulted in the thickening of the macula on SD-OCT in patients with NPDR. Moreover, thinning of the choroidal layer was observed, possibly indicating the vasoconstrictive effect of HBOT on the choroid. This study’s findings supported the hypothesis by Kiryu and Ogura [26], who proposed that direct retinal oxygenation reduces retinal leakage via vasoconstriction.

In contrast, a study conducted by Gün et al. [27] indicated no acute effects on the CMT or choroidal thickness in patients with NPDR and type 2 diabetes who underwent HBOT to treat diabetic foot ulcers. However, their study evaluated only the immediate effect of HBOT on the macular and choroidal thicknesses, and the discrepancy with the results of other studies may be attributed to obtaining measurements before and after the first session of treatment without considering the cumulative effect of therapy.

The pathogenesis of DR involves various inflammatory pathways, such as upregulated chemokines and cytokines. VEGF plays a crucial role in the development of vascular lesions in DR, and it promotes pathological angiogenesis and vascular permeability. Moreover, the severity of DR is highly correlated with the intra-ocular VEGF concentration [28].

Studies conducted using animal models have demonstrated that HBOT upregulates VEGF production to facilitate angiogenesis, which is required for wound healing [29,30]. This may partly explain the effectiveness of supportive HBOT in the treatment of chronic diabetic foot ulcers [31,32]. Based on these data, HBOT could contribute to the deterioration of diabetes owing to an increase in VEGF. However, to our best knowledge, no study has confirmed an increase in VEGF secretion in the vitreous body following HBOT. Furthermore, no study has been conducted on the exacerbation of DR following HBOT, except for two case reports on vitreous hemorrhage [33,34]. In one of these cases, the patient had a history of PDR in both eyes. Active lesions in the left eye were treated with panretinal photocoagulation; however, vitreous hemorrhage occurred in the right eye following vitrectomy. After eight sessions of HBOT to treat a chronic foot ulcer, vitreous hemorrhage occurred in the left eye [33]. In the other case, a patient with a history of bilateral DR treated a few years previously with panretinal photocoagulation received HBOT as an adjunctive treatment for diabetic foot ulcers [34]. Phacoemulsification surgery with intra-ocular lens insertion was performed 3 months and 1 year before HBOT in his left and right eyes, respectively. Ophthalmic examination before the first HBOT session revealed NPDR. Fundoscopy following the fifth session of HBOT revealed pre-retinal hemorrhage of the left eye. Deterioration of the proliferative changes was observed in both eyes after 12 sessions of HBOT. However, the effect of the cataract surgery and vitrectomy must be considered in this patient [34].

### 3.2. HBOT for the Treatment of CRAO/BRAO

The use of HBOT to treat CRAO, an emergency condition that commonly results in severe and irreversible vision loss in the affected eye, is supported by little scientific evidence. Vision loss associated with this condition is painless and typically ranges from light perception to hand motion. The natural course of CRAO is characterized by poor visual outcome and NV of the iris, retina, and optic disc, which occurs in up to 15% of cases [35,36]. To date, no standard treatment has been established for CRAO. Several treatments, such as pentoxifylline, carbogen inhalation, sublingual isosorbide dinitrate, ocular massage with a three-mirror contact lens, acetazolamide, mannitol, methylprednisolone, tissue plasminogen activator, and neodymium: yttrium-aluminum-garnet laser, as well as surgical modalities, such as anterior chamber decompression and pars plana vitrectomy with removal of the embolus, have been suggested [2,37,38,39,40].

The outer retina is supplied by the ciliary arteries via the choriocapillaris, whereas the inner retina is supplied by the central retinal artery (CRA). Sudden vision loss in CRAO results from cell death in the inner retinal layers. The outer layers are comparatively spared; thus, oxygen from the CC may theoretically diffuse to the inner layers of the retina in sufficient quantities to maintain retinal function and restore vision when supplemental oxygen is provided [36]. The retinal vasculature is terminal, without anastomoses between the retinal and choroidal vessels, except when a cilioretinal artery is present, which is observed in approximately 15–30% of the population. When CRAO occurs, the retinal cells can survive for a few hours due to the oxygen present within the vitreous chamber and increased anaerobic glycolysis in the visual cells [2]. This can be achieved with normobaric hyperoxia in some cases, whereas HBOT may be required in other cases. Under normoxic conditions, the CC accounts for the majority (60%) of the oxygen supply to the retina, regardless of whether hyperoxic conditions can fully meet the oxygen requirement of the retina through the CC.

When the arterial blood flow is interrupted, the affected tissue enters a phase of ischemia. Blood flow may subsequently be restored, as observed in the case of arterial occlusions, or the ischemia may persist until the affected cells die and the tissue undergoes necrosis. The period during which the tissue remains ischemic but retains the potential to recover if blood flow is re-established is referred to as the “ischemic penumbra” [41]. The administration of HBOT in animal models of retinal damage has shown promising results in reducing apoptosis. Specifically, studies on experimental CRAO have demonstrated that when HBOT is initiated after the onset of the condition, the rate of apoptosis can be reduced from 58% cell loss to 30% cell loss [42]. Therefore, patients presenting within 24 h of symptom onset might be considered for HBOT.

Although there have been a few case reports on patients presenting after this time interval showing positive results when treated with HBOT, the majority of cases do not respond to treatment beyond this point [6,7,12,17,18,20]. The pertinent physiology suggests that patients with branch RA occlusions and central RVOs may also benefit from HBOT; however, there are insufficient data in the literature to support this as a routine recommendation [6,21,23,26].

The effectiveness of the therapy relies on providing supplemental oxygen as early as possible after the onset of vision loss and before the retinal tissue is irreversibly damaged. However, the main challenge is the maintenance of the retinal activity until circulation is restored via natural recanalization, which may take up to 72 h. The degree of occlusion of the blocked vessel determines the target partial oxygen pressure; therefore, some patients may not respond to HBOT even if it is initiated promptly. The effectiveness of therapy also depends on the location and etiology of the occlusion and the presence or absence of the cilioretinal artery [43].

In the largest published series of patients with CRAO, Hayreh and Zimmerman [43] described the natural progression of this condition without HBOT. Patients with transient CRAO (resolution of symptoms in minutes to hours) and those with cilioretinal arteries had much better outcomes than those who did not. Among the patients without cilioretinal arteries or transient presentations, 80% had a natural outcome of counting fingers, and only 1.5% achieved a final vision of 20/40 or better. The cilioretinal artery, which is present in approximately 15–30% of individuals, is a part of the ciliary (not retinal) arterial supply; however, it supplies the area of the retina around the macula (central vision area). If it is present, central vision may be preserved during CRAO. An adequate partial pressure of oxygen must be maintained to keep the retina viable until circulation is restored via natural recanalization, which usually occurs within 72 h. However, providing sufficient supplemental oxygen early after the onset of vision loss to prevent irreversible retinal damage is challenging.

Rozenberg et al. [44] compared visual outcomes of patients treated for CRAO using HBOT alongside standard care against those receiving standard care alone at various medical centers. They included adults with CRAO symptoms within 24 h. HBOT involved three initial sessions within 24 h, followed by daily sessions. Three patients stopped HBOT early due to ear issues or seizures and nosebleeds. Though groups had similar risk factors and initial eye conditions, the HBOT group, despite being older with shorter symptom duration, showed significantly improved final vision after adjusting for age, sex, and symptom duration. The study suggests adding HBOT to standard care improves final visual outcomes for CRAO, advocating its use in tertiary medical centers.

Similar results were obtained in the experiment by Masters et al. [45] in their retrospective study, which suggested that HBOT implementation in patients with CRAO yields positive results. It was found that 72% of the patients experienced improvement in VA for up to 30 months after treatment. The patients who received treatment within 12 h of symptom onset showed the most significant improvement. Although some patients experienced negative effects, such as middle ear barotrauma and fear of confined spaces, these side-effects did not affect the effectiveness of the treatment.

The results confirm conclusions drawn by Bagli et al. [1], who conducted a study that included 10 patients diagnosed with CRAO who received HBOT. In addition to receiving 20 sessions of HBOT, oral acetazolamide and topical beta-blockers were administered to the patients at the earliest possible time. The average VA was 3.0 before treatment (measured on the logMAR scale) and improved to logMAR 1.8 on average after treatment, and no complications or adverse events were reported.

The study by Lifson et al. [35] investigated the effects of HBOT on VA and NV. They suggest that HBOT may have a protective effect against NV. Moreover, the findings of their study indicate that HBOT may have the potential to enhance long-term VA, and the protective effect of HBOT on NV may lead to better visual outcomes for patients over time.

The study by Elder et al. [46], who identified 31 patients with acute retinal artery occlusion (ARAO) over 10 years, also yielded similar results. All patients underwent at least one HBOT session within 3 to 25.5 h from symptom onset. Thirteen patients also received anticoagulants. After initial improvement with HBOT, seven had good, lasting visual recovery (6/18 or better). Notably, all nine patients who showed permanent improvement were treated within 10 h of symptom onset. Anticoagulation did not significantly relate to permanent recovery. Early HBOT intervention seems beneficial for ARAO, potentially resulting in good visual recovery.

A recent study published in *Undersea and Hyperbaric Medicine* evaluated the effectiveness and safety of a standardized protocol for HBOT in patients with RAO [2]. A total of 28 patients with CRAO or branch RAO (BRAO) received urgent 90-min HBOT sessions as initial treatment, followed by two daily sessions for at least 15 days. Reperfusion was assessed via fluorescein angiography (FA) at day 15; if absent, treatment continued for another week with a subsequent assessment at day 21. The primary goal was a ≥0.3 logMAR VA improvement at 1 month, achieved by 50% of patients. No major HBOT-related side-effects were reported. Monitoring was via retinal FA guided treatment duration for better revascularization oversight. Additionally, the study noted recent onset cerebral stroke in three (10.7%) RAO patients.

Similar results were obtained in the observational study by Kim et al. [47] that examined the effect of adjunctive HBOT on VA in adult patients with CRAO symptoms. Among the 19 patients, 10 received HBOT treatment, whereas the remaining patients did not. The HBOT group showed a significantly greater improvement in VA.

Another retrospective study included 13 patients with CRAO or BRAO [48,49]. Clinically significant visual improvement was seen in 55.5% with CRAO and 75% with BRAO, defined as a decrease in logMAR of 0.3. Initial treatments involved normobaric oxygen, hypotensive medication, eye massage, and aspirin. Patients received two daily 90-min HBOT sessions for 3 consecutive days, with additional sessions if visual acuity continued to improve. Early treatment within a median of 9 h from symptom onset seemed to be correlated with better outcomes.

Schmidt et al. [8] compared two groups of non-arteritic retinal BRAO patients in a non-randomized retrospective study: one received HBOT, the other hemodilution alone (control). Both groups had 14 patients with matched initial VA levels. At discharge, final VA in the HBOT group was 0.69 ± 0.29, significantly better than the control group’s 0.32 ± 0.23. The study suggests significant VA improvement with HBOT, proposing its potential as a rescue therapy during early BRAO stages until potential reperfusion occurs.

In a controlled, non-randomized trial by Menzel-Severing et al. [50], 51 patients received HBOT and hemodilution, while 29 received hemodilution alone for ARAO. The HBOT group showed a mean VA improvement of three lines compared to one line in the hemodilution-only group, though this difference was not statistically significant (*p* < 0.0001). At discharge, 38.0% in the study group gained three lines or more compared to 17.9% in the control (*p* = 0.06), and at follow-up, proportions were 35.7% versus 30.8% (*p* = 0.76), respectively.

Numerous studies have reported cases of eyesight restoration in patients with CRAO following HBOT. The study by Kim et al. [51] offered an analysis of a case of an 81-year-old woman who presented with sudden vision loss in her right eye, with ‘hand motion’ VA. A 10-h delay occurred in receiving therapy. Although she received oxygen via a facial mask, no substantial improvement was observed. Since anterior chamber paracentesis did not improve vision, she received HBOT for three sessions over 3 days. VA improved to 0.4 in the affected eye and remained stable after discontinuing HBOT. VA was maintained at 0.8 without complications 1 month after discharge.

In a different case, a healthy 48-year-old woman experienced sudden blurred vision in her left eye, present for 2 days before admission. She had prior hormonal treatment. With non-ischemic cilioretinal artery occlusion confirmed via FA, she underwent 30 daily 2-h sessions of HBOT. The treatment succeeded, improving her left eye’s VA to 10/10 (20/20) [48].

In Butler et al.’s [52] case, a 71-year-old with CRAO initially responded well to HBOT 9.5 h post-vision loss but saw vision decline again soon after. Subsequent HBOT sessions had less effect due to scheduling delays. Even though the first response suggested no permanent retinal damage, recurrent vision loss indicated ongoing issues. They recommended immediate referral to a stroke center for those responding well to initial HBOT, with close visual monitoring. For recurring vision loss, they proposed using intermittent 100% normobaric and hyperbaric oxygen aggressively until recanalization. This study outlines a management plan based on these findings.

Gokce et al. [53] describe a case of a healthy 48-year-old woman with sudden vision blurring in her right eye, ongoing for 2 weeks. She had recently been at high altitude (2540 m/8333 feet) but had no health issues there. On admission, her right eye VA was 10/20, and left eye was 10/10, suggesting non-ischemic CRVO. Undergoing 2-h daily HBOT for 11 days, her right eye’s VA improved to 20/20, and the visual field scotoma vanished post-treatment. The sessions concluded successfully without complications or other medical incidents.

Wu et al. [39] conducted a meta-analysis involving seven randomized controlled trials with 251 individuals receiving various oxygen therapies for RAOs. Most HBOT studies had low bias risk and showed significant visual improvements when combined with other treatments. However, the independent effect of HBO alone on visual outcomes remains uncertain. Oxygen therapy, especially HBOT, led to a statistically significant increase in VA compared to non-oxygen therapy groups, regardless of treatment timing after symptom onset. The study suggests that combining oxygen therapy, particularly HBOT, with other treatments can benefit visual outcomes. Assessing therapy effectiveness via the median number of HBOT sessions, typically around 10.5 h, aligns with their findings. Their meta-analysis highlights > 9 h of treatment duration with a median of three HBOT sessions as the most effective.

Youn et al. [54] highlighted the lack of consensus in treating CRAO through a national survey in the United States. The study surveyed directors of vascular neurology, neuro-ophthalmology, and retina fellowship programs at teaching hospitals, revealing significant variation in acute CRAO management among 45 institutions. Only 20% had a standardized approach; treatment providers varied from ophthalmologists to neurologists. While fibrinolysis was offered by 53% of institutions, others provided different treatments or none at all. Carotid imaging was common (89%), but screening for inflammatory states was low (27%). Physicians referring CRAO cases to the emergency department tended to screen more thoroughly for vascular risk factors. The patient population showed an increased risk of cerebrovascular and cardiovascular events, but screening practices varied widely, and some physicians did not routinely refer patients for urgent evaluation and HBOT. Furthermore, studies on RAO were complicated by simultaneous use of multiple treatments alongside HBOT, making it uncertain whether the observed outcomes were solely due to HBOT (Table 1 and Table 2).

### 3.3. Impact of HBOT on Central Corneal Thickness

The corneal endothelium plays a vital role in maintaining stromal hydration and corneal transparency. Hydration and stromal acidosis of the cornea increase, and increased central cornea thickness (CCT) occurs when the oxygen tension of the cornea is reduced due to the use of contact lenses. Alterations in the corneal structure can lead to refraction errors and reduced VA. Hypoxia and high-altitude exposure have been shown to increase the corneal thickness. HBOT has been suggested as a treatment for corneal edema [55]. Ayata et al. [56] investigated the impact of HBOT on CCT. It was found that CCT was considerably decreased in non-diabetic patients after a single HBOT session [56], thereby suggesting that diabetes impairs the function of the corneal endothelial by reducing the Na^+^/K^+^-ATPase activity in diabetic rats [57]. Since this enzyme is the main component of the endothelial pump, impairment of the activity of the enzyme prevents the reduction of corneal edema and slower recovery from hypoxic edema in diabetics compared with healthy individuals. However, this study examined the CCT after only one session of HBOT. Therefore, the obtained data may not be representative of repeated treatments, so further studies on the subject are needed.

### 3.4. The Treatment of Optic Neuropathy with HBOT

Malik et al. [58] reported that prompt treatment with HBOT combined with oral corticosteroids can preserve VA in patients with unilateral or asymmetric bilateral radiation optic neuropathy. The results of four patients with a history of malignant disease who received radiation doses above the accepted limit were summarized in this study. In cases of radionecrosis, oxygen levels are often insufficient to support angiogenesis. Thus, the use of hyperbaric therapy, which delivers nearly 100% oxygen, is justified to disrupt the ongoing ischemic necrosis, and enhance fibroblastic activity, collagen synthesis, and angiogenesis in the radiated tissues [49].

Another case report highlights the use of HBOT as a useful alternative treatment for direct traumatic optic neuropathy (TON), especially when it occurs in combination with traumatic brain injury [59]. A 57-year-old woman with direct TON and contusion brain injury received HBOT for 100 min five times a week for a total of 61 sessions. After receiving delayed HBOT, her VA improved from hand motion to 6/60. In addition, an improvement in the visual field and color vision was also observed.

Interestingly, a similar effect of HBOT was also observed in case reports of successful treatment of postoperative posterior ischemic optic neuropathy [60]. An elderly woman experienced temporary binocular vision loss after receiving an intravenous dose of hydralazine during an angiographic procedure, which caused a transient decrease in blood pressure. She was transferred to a specialized reference center and treated with HBOT according to the decompression sickness protocol. Her vision improved considerably after the first session and continued to improve throughout the course of treatment until completion. Follow-up visits showed that her vision was close to baseline.

Similar findings were reported in another case of traumatic optic neuropathy [61]. Baseline examination of the right eye after injury showed no light perception, afferent pupillary defect, normal anterior segment, and unremarkable dilated fundoscopic examination. Immediate HBOT combined with high-dose steroid therapy and minocycline were initiated. The patient’s vision improved to 20/40 after six sessions of HBOT and intravenous therapy.

### 3.5. Choroidal Neovascularization

Malerbi et al. [62] evaluated the effect of HBOT on choroidal NV. In their study, seven patients received 10 daily 120-min sessions of HBOT. After the sessions, five patients received intravitreal injections of bevacizumab. The mean follow-up period was 150 days. At the end of follow-up, the CNV area and diameter decreased in five patients, remained the same in one patient, and increased in one patient. The authors suggested that HBOT, either as a monotherapy or in combination with intravitreal anti-VEGF agents, may serve as a treatment option for patients with active CNV, potentially delaying its progression. However, owing to the small number of participants enrolled in the study, the short follow-up period, lack of a control group, and inclusion of only participants with poor prognostic features at baseline, the data remain unconvincing.

### 3.6. Case Reports of HBOT Use

One case report [63] suggested that HBOT might be beneficial as a rescue treatment for mumps retinitis and retinal vasculitis owing to its potential neuroprotective, anti-inflammatory, and antiapoptotic effects. A 4-year-old boy was admitted to the emergency department with a 2-day history of sudden vision loss in both eyes. The patient had a history of fever and painful bilateral parotid swelling 1 month earlier. His VA was limited to light perception. The patient was unsuccessfully treated with intravenous acyclovir, along with topical and oral steroids. HBOT, consisting of a 1-hour daily session of direct respiration in a cabin, was initiated 4 days after the presentation, and the patient received 40 HBOT sessions. One month after the initial presentation, BCVAs were 20/100 in the right eye and 20/320 in the left eye. SD-OCT revealed the progressive recovery of the outer retina in the right eye and persistent outer retinal layer damage in the left eye.

HBOT may be an effective treatment for periorbital and orbital emphysema owing to its ability to accelerate the removal of nitrogen from the adipose tissue. A patient with orbital emphysema successfully treated using HBOT has been reported [64]. A 40-year-old patient with a history of retinal detachment presented to the emergency department with pain, decreased eye motility, and edema of the upper eyelid. Clinical examination revealed periorbital crepitus; therefore, soft tissue decompression was performed immediately using a small-gauge needle. However, orbital emphysema recurred shortly after gas aspiration, suggesting that the gas was trapped in the soft tissue. Therefore, HBOT was implemented. The patient received 35 HBOT sessions until the clinical symptoms resolved, and the gas was completely reabsorbed from the retrobulbar space. The effectiveness of the therapy was determined by accelerating the elimination of nitrogen from the adipose tissue, leading to shrinkage of the gas bubble in the periocular space, thereby avoiding further irreversible changes in structures such as the optic nerve.

HBOT is also recommended for the treatment of acute carbon monoxide (CO) poisoning, ideally starting within 6 h. It reduces cognitive recurrence 6 weeks after acute CO poisoning. HBOT also prevents delayed neurological symptoms and is used to treat them. Senol et al. [10] reported the case of a 21-year-old female who presented with epilepsy and vision loss as a late consequence of CO poisoning. Four years after the poisoning episode, her VA was 0.2 in both eyes. A positron emission tomography (PET) scan revealed reduced metabolism in both posterior temporal and occipital lobes. Ophthalmologic examination showed no abnormalities in the anterior visual pathways. The patient received 50 HBOT sessions in a hyperbaric chamber at 2.4 ATA for 120 min daily over 3 months. The VA improved by three lines on the Snellen chart, from 0.2 to 0.5, in both eyes, and the patient regained her ability to read. Moreover, subsequent PET scans revealed increased metabolism in the posterior temporal and occipital lobes after HBOT. No epileptic seizures or other complications of HBOT were observed during therapy.

The authors of another paper reported the administration of HBOT as adjuvant therapy for acute macular neuroretinopathy in systemic lupus erythematosus in conjunction with immunosuppression in two patients [11]. After 12 cycles of HBOT, functional and anatomical improvements were obtained in ophthalmic examination and were maintained over more than 1 year of follow-up, and visual field scotoma showed complete resolution.

## 4. Disadvantages of HBOT

Breathing pressurized oxygen causes modest changes to the saturation level of hemoglobin while causing substantial amounts of unburnt oxygen to remain in the tissues. These oxygen levels may exceed those required by the tissues or their capacity to use the available oxygen [65]. Exposure to oxygen at high pressures can increase the transfer of oxygen into the tissues by up to 20 times the normal levels. HBOT leads to an increase in the levels of reactive oxygen or nitrogen species in the tissues and blood, which may lead to oxidative DNA damage.

McMonnies [65] reported that HBOT increases the oxygen pressure and concentration of reactive oxygen species in the blood, which may theoretically contribute to complications of HBOT, such as dry eye, cataracts (only observed in patients receiving more than 150 total hours of treatment), age-related macular degeneration, reversible myopic shift (up to −4.50 diopters), and keratoconus [66]. Less frequent but more severe adverse events include otic barotrauma, reversible bronchopulmonary toxicity, and seizures [58]. Although high-pressure oxygen therapy can have beneficial effects on biochemistry, cellular function, and physiology by significantly enhancing oxygen transfer into the tissues, the aging eye may be particularly vulnerable to excess reactive oxygen species in the tissues when antioxidant activity is deficient. The prevalence of complications may be underestimated if they are mistaken for age-related changes. Age-related macular degeneration is typically linked to oxidative stress and the death of retinal pigment epithelial cells under normal circumstances, and according to the author [58], HBOT may exacerbate these processes. Myopia may occur as a direct toxic effect of oxygen on the crystalline lens, and since HBOT can induce reversible myopia, all keratorefractive surgeries should be postponed unless otherwise indicated. These conclusions are consistent with the results obtained by Riedl et al. [67], who studied myopic shift and lens opacification following HBOT. The authors provided evidence that myopic changes are common but transient complications of HBOT. Long-term exposure to intensive HBOT has also been linked to the development of nuclear cataracts, which are more prevalent in older patients. The cataract-related changes observed in this study progressed linearly and showed no signs of regression after treatment, indicating the risk of decreased lens transparency as a consequence of HBOT.

In addition to age-related macular degeneration and keratoconus, HBOT-induced oxidative stress may have significant adverse effects on other ocular diseases [68]. Therefore, careful examination and evaluation of the potential risks and benefits of this form of therapy are recommended for all patients. Dietary antioxidant supplementation may be indicated in the presence of comorbidities that are affected adversely by oxidative stress. The implementation of HBOT may need to be modified or even contraindicated in these cases. Therefore, close monitoring of eye health and any pre-existing eye diseases is recommended.

McMonnies [68] also emphasized the potential disadvantages of HBOT in the treatment of patients with coexisting glaucomatous pathology, as oxidative stress involvement in optic disc neuropathy is suspected. However, if the implementation of HBOT is essential, it should be administered cautiously, and the patients should be monitored progressively for glaucoma progression. The increased partial pressure of oxygen in the anterior chamber angle can damage trabecular meshwork cells [69]. The risk of damage to the trabecular meshwork cells may be higher if hyperbaric oxygen is delivered by a hood that exposes the anterior ocular surface to higher-than-normal oxygen levels. The use of oronasal mask delivery of HBOT appears to be more appropriate in such cases [68].

The exacerbation of macular edema, probably associated with HBOT, was also reported in one case report. Acute and severe macular edema 2 days after the seventh session of HBOT was observed in a patient with a ruptured retinal arterial microaneurysm or macroaneurysm [70]. 

**Figure 1:**
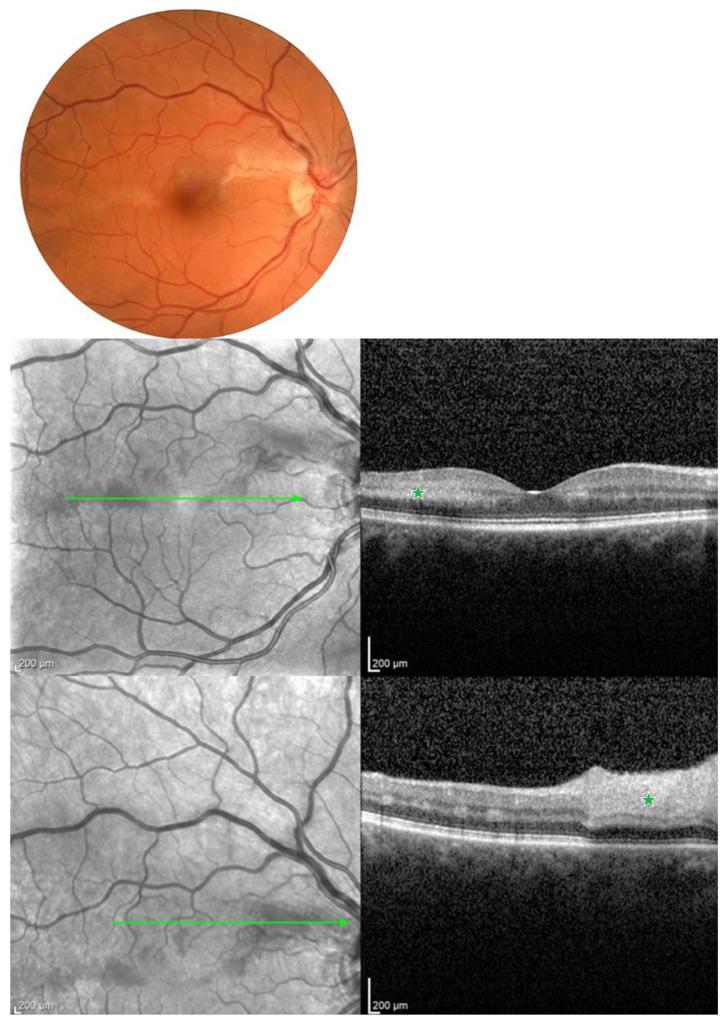
Color fundus photography and spectral-domain (SD-OCT) of 42-year-old patient with hemiretinal artery occlusion (HRAO) and paracentral acute middle maculopathy (PAMM). The first row shows a color fundus photography illustrating irregular zones of ischemic whitening in the macular region. In the second row of the cross-sectional OCT demonstrating parafoveal inner nuclear layer (INL) hyperreflectivity (green asterisk), the third row demonstrates thickening and hyperreflectivity of the inner and middle retinal layers (green asterisk) indicating the presence of substantial retinal ischemia. Green arrow indicate the area of ischemia.

**Figure 2:**
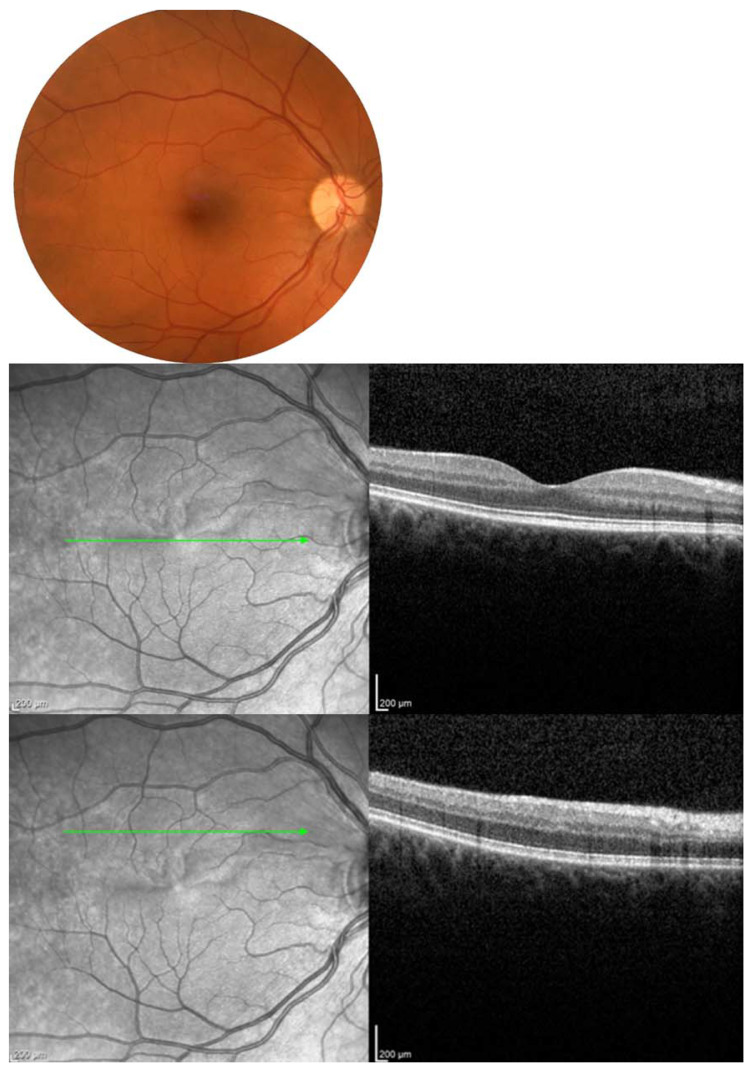
After 15 days of hyperbaric chamber therapy, the color fundus photography shows reperfusion of the previously mentioned ischemic areas with corresponding OCT images also demonstrating a decrease in retinal thickness and enhancement in morphological parameters of the retina. Green arrow indicate the area of ischemia.

**Figure 3:**
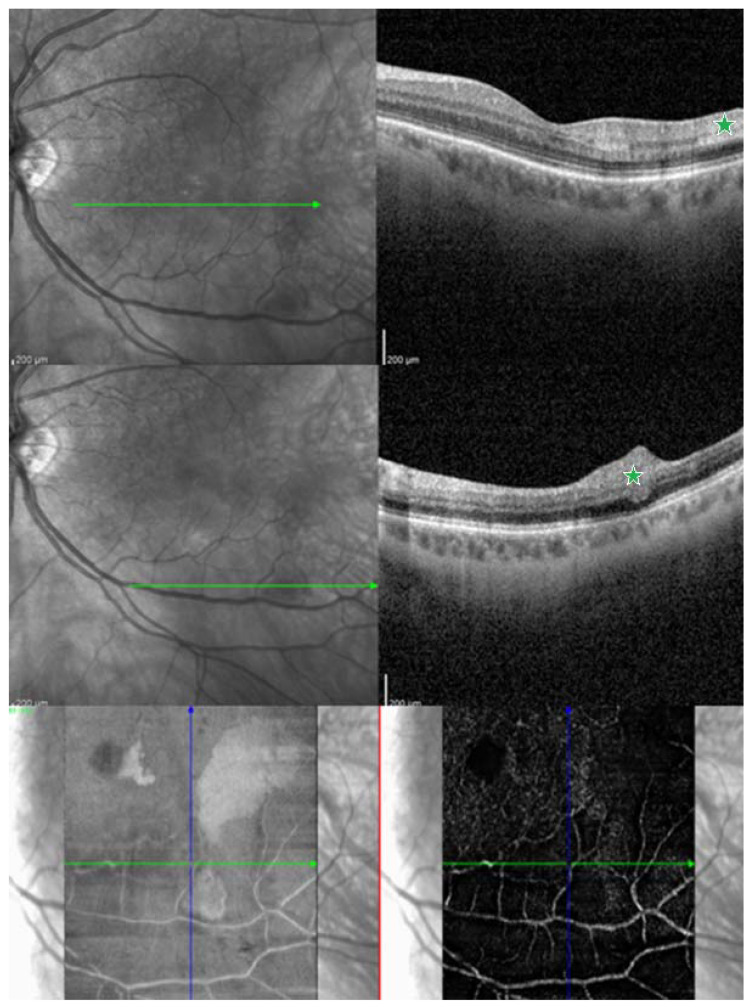
Shows a spectral-domain optical coherence tomography (SD-OCT) and optical coherence tomography angiography (OCTA) of a 56-year-old patient presenting with a combination of cotton wool spot and paracentral acute middle maculopathy (PAMM) as a result of branch retinal artery occlusion (BRAO)). The first row shows an OCT through the parafoveal region, which reveals the inner nuclear layer (INL) hyperreflectivity in a band-like pattern (green asterisk). In the second row, an OCT through the inferior region of macula indicates infarction in the inner retinal layers, cotton wool spot (green asterisk). On the third row, the OCTA shows impaired perfusion in the intermediate capillary plexus (ICP), a component of the deep vascular complex (DVC), temporally from the fovea, corresponding with the PAMM lesion observed on OCT. Green arrow indicate the area of ischemia.

**Figure 4:**
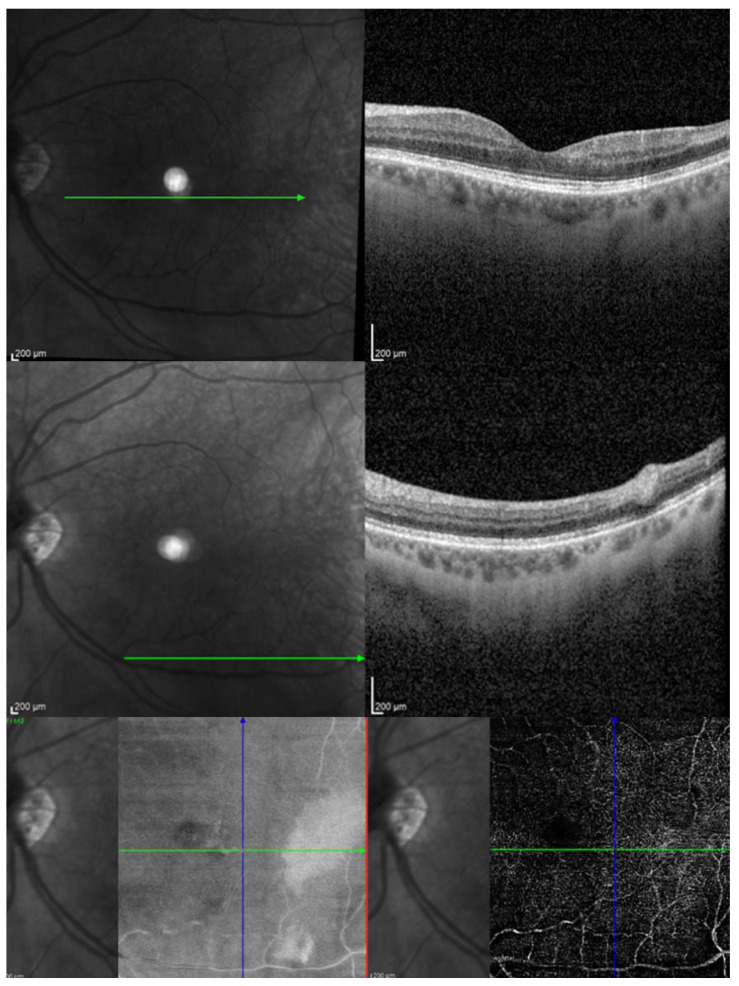
After 15 days of hyperbaric chamber therapy, the OCT reveals a reduction in retinal thickness and OCTA shows improvement in retinal circulation.

The authors present the image of patients from own observation (Figure 1, Figure 2, Figure 3 and Figure 4).

## 5. Discussion

Analysis of the different indications for HBOT has provided the best body of evidence to indicate its effectiveness in CRAO/BRAO. Some evidence suggests that HBOT may be the only effective treatment modality for the management of CRAO among several treatment modalities tested over the last few years. The traditional therapeutic regimens for CRAO, including anterior chamber paracentesis, ocular massage, intraocular pressure-lowering medications, and vasodilators [71], aim to promote the downstream movement of the embolus. However, these treatments typically do not prevent severe and permanent vision loss, even when applied promptly, and they cannot be considered the gold standard for the treatment of CRAO/BRAO [43,72].

The ideal time from the onset of CRAO/BRAO to the initiation of HBOT remains unclear. Although it is understood that HBOT should be initiated as early as possible, the exact timeline for initiating treatment and the timeline of irreversible anoxic retinal damage in humans remains unclear. Some authors have suggested a duration of 6–6.5 h; however, the residual blood flow of the retina differs significantly among patients, which may explain the high variability in visual results, regardless of the treatment time delay [73]. Thus, the treatment time is not a reliable predictor of treatment effectiveness, and clinicians should consider a time limit for HBOT implementation in patients with CRAO. Similarly, the optimal number of sessions that ensure improvement without causing adverse events is also unclear. Many studies have shown favorable results for the implementation of HBOT, and at least eight treatments are generally recommended [69,74].

The existing literature on the application of HBOT in ophthalmology primarily comprises case reports and observational studies, with a paucity of high-quality randomized controlled trials. The majority of available research falls into the lower tiers of the evidence-based medicine (EBM) pyramid, reflecting the limited methodological rigor inherent in these study designs. While case reports can provide valuable insights into specific clinical scenarios, they often lack generalizability and are susceptible to biases. Observational studies, though informative, may be confounded by various factors, limiting their ability to establish causal relationships.

Furthermore, the existing literature on hyperbaric oxygen therapy in ophthalmology is plagued by several methodological shortcomings that undermine the reliability and generalizability of its findings. A recurrent issue across studies is the use of inadequately small sample groups, which hampers the statistical power and external validity of the research. Moreover, the disparity in the time elapsed from onset to treatment among the studied groups introduces a confounding variable, making it challenging to draw conclusive insights into the true efficacy of HBOT. Moreover, these studies had several limitations, such as lack of a standardized HBOT protocol, a subjective main outcome measure, and several other confounding factors. To date, there is no consensus on the duration of HBOT and the ideal number of daily sessions. However, before considering HBOT as an effective treatment for RAO, it is also essential to compare its results with the natural course of the disease.

The prevalence of case reports in the literature is another notable limitation, suggesting a scarcity of robust experimental designs. Additionally, the lack of standardized methods for initial treatment, the absence of sex- and age-matched control groups, and the oversight of comorbidities collectively compromise the internal validity of these studies. Addressing these methodological discrepancies is imperative for advancing the understanding of HBOT’s role in ophthalmology and establishing evidence-based practice, Table 3.

To establish a solid foundation for evidence-based practice in ophthalmic HBOT, future studies must prioritize larger, well-defined patient cohorts, employ standardized methodologies, and meticulously account for the unique characteristics of different patient subgroups. Only through such methodological rigor can the field advance beyond anecdotal observations and anecdotal reports, providing clinicians with reliable guidance for the integration of HBOT into ophthalmic care. Moreover, there is a pressing need for the development of standardized algorithms for the treatment of CRAO and BRAO. Establishing such guidelines would not only contribute to the homogeneity of study methodologies but also provide clinicians with a structured and evidence-based approach to managing these sight-threatening conditions, ultimately enhancing the quality of patient care.

Studies have shown that HBOT is cost-effective, with the average cost per HBOT session estimated at approximately USD 500 (EUR 325) [72]. Thus, a series of five treatments would amount to approximately USD 2500, which is comparable with the cost of a cataract operation provided by the New Zealand government at the time (approximately NZD 4000). ARAO treatment with HBOT is one of the most cost-effective interventions. In high-level Cochrane analysis, the percentage of useful vision recovery (nine of 31 patients, 29%) following HBOT (8%, a conservative estimate), the number of patients requiring treatment with HBOT for useful visual recovery was approximately five, indicating that HBOT is a cost-effective intervention.

In the future, the effect of HBOT should be evaluated via experimental research on an animal model.

Shanshan et al. attempted to investigate the promotion of corneal epithelial wound healing after injury by administering increased oxygen concentration through inhalation or goggles [75]. It was found that local oxygen supply through goggles had the most beneficial effect, and acetylcholine may play a substantial role in the healing process. Thus, it was suggested that oxygen enhances the release of cytokines and growth factors to promote tissue healing, whereas acetylcholine promotes corneal epithelial wound healing through a series of synaptic transmissions and transmembrane transport [75].

## 6. Conclusions

Before implementing HBOT, detailed risk assessments must be conducted to consider the possible side-effects, such as barotrauma, ear pain, tympanic membrane rupture, and generalized oxygen toxicity seizures in the central nervous system. Compiling evidence on the effectiveness of HBOT and elaborating on the available literature could expedite the implementation of official recommendations for the use of HBOT. RAO is the only indication for HBOT for which a considerable amount of literature has been published. Hyperbaric oxygen treatment, where available, is a safe, low-cost, and moderately effective treatment option for patients with ARAO. A multicenter, randomized controlled trial of HBOT is feasible but would be logistically difficult. Currently, there are no good treatments for RAO, and we need to evaluate any treatments that show promise in a rigorous manner. Future studies are necessary to determine further indications, contraindications, and schemes. Animal models may be helpful in expanding the pathophysiological basis; moreover, high-quality studies resulting in meta-analyses may result in wider use of HBOT in vision-threatening diseases.

## Figures and Tables

**Table 1:** Existing literature.

Report	CRAO/BRAO	Study Objective	Number of HBOT Sessions	Conclusions/Findings
Rozenberg et al. [44]	CRAO	Comparing HBOT vs. standard care (SC) outcomes.	Three sessions within 24 h, then daily until max VA reached	HBOT group had markedly better final VA than that of controls
Masters et al. [45]	CRAO	Reporting outcomes from patients treated with HBOT.	Total of 10 sessions	72% showed improved vision up to 30 months post-treatment, notably higher in those treated within 12 h of symptom onset
Bagli et al. [1]	CRAO	Presenting visual outcomes in HBOT-treated patients	Total of 20 sessions	Initial average logMAR 3.0; Improved to logMAR 1.8 post-treatment
Lifson et al. [35]	CRAO	To assess enhancement in VA and reduction in neovascularization post-HBOT	20% of patients developed neovascularization after HBOT compared to 29.8% of those who did not undergo HBOT (*p* < 0.05).	HBOT exhibits significant protection against neovascularization, potentially enhancing long-term visual acuity
Elder et al. [46]	CRAO/BRAO	Utilizing HBOT for acute retinal artery occlusion treatment	At least one session	Early HBOT intervention benefits ARAO patients, fostering potential visual recovery.
Vincenzo et al. [2]	CRAO/BRAO	To evaluate the effectiveness and safety of a standardized HBOT protocol	Two sessions daily for at least 15 days	50% of patients achieved an improvement in VA of at least 0.3 logMAR
Kim et al. [47]	CRAO	To examine the effect of adjunctive HBOT on VA on adult patients	19 were included in the study, of which, 10 patients (52.6%) were treated with adjunctive HBOT	HBOT group showed a significantly greater improvement in VA compared to that of the control group
Lopes et al. [49]	CRAO/BRAO	To evaluate the efficacy and safety of HBOT	Two sessions daily for 3 days, then based on BCVA improvement till stability	Early HBOT improved BCVA outcomes post-symptom onset
Schmidt et al. [8]	BRAO	Comparing HBOT vs. standard care (SC) outcomes.	5 times within 48 h, with 3 treatments within the first 24 h	HBOT patients showed significant VA improvement versus control
Menzel-Severing et al. [50]	CRAO/BRAO	Comparing HBOT + hemodilution vs. hemodilution alone effects.	Five treatments in 48 h, three within the first 24	HBOT saw a 3-line VA improvement versus 1 line in hemodilution alone; not statistically significant
Wu et al. [39]	CRAO/BRAO	To examine seven randomized controlled trials using various types of oxygen therapy	-	Oxygen therapy, especially HBOT, may offer visual advantages when paired with other treatments.

Note: see full graphs of patient results in original papers.

**Table 2:** Case reports.

Report	CRAO/BRAO/CRVO/CLRAO	Therapy	No. of HBOT Sessions	Delay to Tx	Initial VA	Final VA
Kim et al. [51]	CRAO	HBOT, ocular massage, topical brimonidine and dorzolamide/timolol	3 over 3 days	10 h	hand motion	0.4 (OD) for far vision; 0.5 (OD) for near vision
Khallouli et al. [48]	CLRAO CRVO	HBOT	total of 30 sessions	2 days	7/10	10/10
Butler et al. [52]	CRAO	HBOT, ocular massage, timolol drops, and acetazolamide	Total of 7 sessions	9.5 h	hand motion	Count fingers
Gokce et al. [53]	CRVO	HBOT	Total of 11 sessions	2 weeks	10/20	20/20

Note: see full graphs of patient results in original papers.

**Table 3:** Limitations of existing studies on HBOT.

Drawbacks of existing studies of HBOT
Primarily comprises case reports and observational studies
Paucity of high-quality randomized controlled trials
Limited methodological rigor inherent in these study designs
Small sample groups
Absence of sex- and age-matched control groups
Disparity in the time elapsed from onset to treatment among the studied groups
Lack of standardized HBOT protocol
Lack of consensus on the duration of HBOT and the ideal number of daily sessions
Oversight of comorbidities

## Data Availability

All the data are available in public online.
